# Squalene attenuates the oxidative stress and activates AKT/mTOR pathway against cisplatin-induced kidney damage in mice

**DOI:** 10.3906/biy-1902-77

**Published:** 2019-06-13

**Authors:** Arzu SAKUL, Mehmet OZANSOY, Birsen ELİBOL, Şule AYLA, Mehmet Yalçın GÜNAL, Yasemin YOZGAT, Hüveyda BAŞAĞA, Kazım ŞAHİN, Rümeyza KAZANCIOĞLU, Ülkan KILIÇ

**Affiliations:** 1 Department of Medical Pharmacology, School of Medicine, İstanbul Medipol University, İstanbul, Turkey; 2 Department of Physiology, School of Medicine, İstanbul Medipol University, İstanbul, Turkey; 3 Department of Medical Biology, Faculty of Medicine, Bezmialem Vakıf University, İstanbul, Turkey; 4 Department of Histology and Embryology, School of Medicine, İstanbul Medipol University, İstanbul, Turkey; 5 Department of Physiology, Alanya Alaaddin Keykubat University School of Medicine, Antalya, Turkey; 6 Regenerative and Restorative Medical Research Center (REMER), İstanbul Medipol University, İstanbul, Turkey; 7 Biological Sciences and Bioengineering Program, Faculty of Engineering and Natural Sciences, Sabancı University, İstanbul, Turkey; 8 Department of Animal Nutrition, Faculty of Veterinary Medicine, Fırat University, Elazığ, Turkey; 9 Department of Nephrology, Faculty of Medicine, Bezmialem Vakıf University, İstanbul, Turkey; 10 Department of Medical Biology, Faculty of Medicine, University of Health Sciences, İstanbul, Turkey

**Keywords:** AKT, cisplatin-induced nephrotoxicity, mice, mTOR, oxidative-stress, squalene

## Abstract

The clinical use of cisplatin, which is a first-line anticancer agent, is highly restricted due to its adverse effects on kidneys that lead to nephrotoxicity. Therefore, some potential reno-protective substances have been used in combination with cisplatin to cope with nephrotoxicity. Due to its high antitumor activity and oxygen-carrying capacity, we investigated the molecular effects of squalene against cisplatin-induced oxidative stress and kidney damage in mice. Single dose of cisplatin (7 mg/kg) was given to male Balb/c mice. Squalene (100 mg/kg/day) was administered orogastrically to mice for 10 days. Following sacrification, molecular alterations were investigated as analysis of the levels of oxidative stress index (OSI), inflammatory cytokines and cell survival-related proteins in addition to histopathological examinations in mice kidney tissue. The level OSI and Interferon-gamma (IFN-γ) decreased in the cisplatin and squalene cotreated mice compared to cisplatin-treated mice. Squalene treatment also increased the activation of protein kinase B (AKT). Furthermore, cisplatin-induced inactivation of mammalian target of rapamycin (mTOR) and histopathological damages were reversed by squalene. It may be suggested that squalene ameliorated the cisplatin-induced histopathological damages in the kidney through activation of AKT/mTOR signaling pathway by regulating the balance of the redox system due to its antioxidative effect.

## 1. Introduction

**Figure 1:**
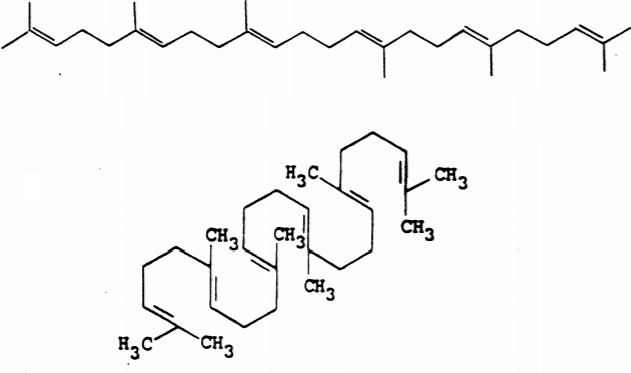
Chemical structure of squalene; a simple linear mode of presentation and the cyclization of squalene (Adapted from Newmark, 1999).

Chemotherapeutic agents used in the treatment of various types of cancer have side effects such as ototoxicity and nephrotoxicity. The toxic effects of these antineoplastic drugs can be ignored because of their potency of improving an individual’s chances of survival by impairing mitosis of fast-dividing cancer cells (Kilic et al., 2015a). In general, in terms of their mechanism of action, the chemotherapeutic agents fall into several categories including alkylating agents, antimetabolites, mitotic inhibitors, antibiotics, enzymes, hormones, and hormone antagonists. Cisplatin, a platinum compound (cis diamminedichloridoplatinum(II) (CDDP), is the first member of these chemotherapy drugs which is used against various forms of solid cancers (e.g., small cell lung cancer, squamous cell carcinoma of the head and neck, bladder cancer, cervical cancer and ovarian cancer), sarcomas, and lymphomas (Ho et al., 2003; Li et al., 2017). The action mechanism of cisplatin occurs by binding to DNA of fast proliferating cells and causing the DNA strands to crosslink that triggers cell death in a programmed manner (Wang and Lippard, 2005). However, due to its strong electrophilic nature, cisplatin induces oxidative stress producing adverse side effects such as nephrotoxicity (Arany and Safirstein, 2003; Kilic et al., 2015a). The nephrotoxicity of platinum-class chemotherapeutics can be ameliorated using some free radical scavenging agents (Shimeda et al., 2005; Kilic et al., 2013). Therefore, variety of compounds obtained from medicinal plants is used as chemopreventive antioxidant agents.**It has been documented that the activators of some antioxidant proteins like nuclear factor erythroid 2-related factor 2 (Nrf2), a redox-sensitive transcription factor, such as polyphenols, sulfur-containing terpenoids, carotenoids, and selenium induce the expression of cytoprotective proteins in an antioxidant response elements-dependent manner (Sies and Masumoto, 1997; Kilic et al., 2013). Squalene (2,6,10,15,19,23-hexamethyl-6,6,10,14,18,20-tetracosahexane), a natural 30-carbon organic compound, may be one of these chemopreventive agents that shows beneficial effects on health (Smith, 2000; Sotiroudis and Kyrtopoulos, 2008) (Figure  1). It is a polyunsaturated triterpene which is found in high concentrations in shark liver, vegetable oils, and in the stomach oil of birds (Liu et al., 1976; Vazquez et al., 2008). Previous animal studies proposed that squalene have the antitumor activity and the ability to reduce the cancer risk (Newmark, 1997; Sotiroudis and Kyrtopoulos, 2008). The inhibitory effect on cancer promotion and the selective cytoprotective effect in the protection of normal cells against toxicity of chemotherapeutics while showing no protective efficacy for tumor cells made squalene possible as a primarily used supportive therapy in a variety of cancers (Senthilkumar et al., 2006a and 2006b). The inhibition of Ras farnesylation, the modulation of carcinogen activation and antioxidative activities are the proposed mechanisms for the chemopreventive activity of squalene (Newmark, 1997; Smith, 2000). Squalene attenuates oxidative DNA damage in human cells by reducing the levels of reactive oxygen species in vitro (Warleta et al., 2010). Oxygenation effect of squalene on cells is thought to be effective because higher cellular oxygen levels provide a more efficient metabolic process and thus enhance cell energy metabolism. In addition to its chemopreventive activity through its antioxidant effect, much more research is clearly needed to determine cytoprotective effect of squalene against side effects of chemotherapeutics. Therefore, we aimed to investigate the molecular mechanism of renoprotection of squalene in an animal model of cisplatin-induced kidney damage.

## 2. Methods

### 2.1. Animal experiments

Balb/c mice that are more susceptible to disease models (Foerster et al., 2017) (n = 7 for each group) weighing 25 g obtained from İstanbul Medipol University Research Center (MEDITAM) were used in this study. Because of the protective role of estrogen in the cisplatin-induced kidney damage (Nematbakhsh et al., 2017; Pezeshki et al., 2013), male mice were used in the present study to obtain an effective cisplatin-induced nephrotoxicity model. The animals were acclimatized for 1 week before the study and were maintained under standard conditions (12 h light/dark cycles, 22 °C, and 60% humidity) with ad libitum food and water. All experimental procedures were approved by the Ethics Review Committee for Ethics in Animal Experiments of the İstanbul Medipol University (14.01.2015/05) and guidelines for the Care and Use of Laboratory animals were strictly followed. The mice were divided into five groups; control receiving saline (i.p.) (Cs), control receiving corn oil (250 mg/kg body weight, daily orogastric gavage) (Cco), cisplatin-treated (single dose, 7 mg/kg body weight., i.p. dissolved in 0.9% saline) (Cis), squalene-treated (100 mg/kg squalene (Das et al., 2008) dissolved in warm corn oil, daily orogastric gavage) (Squa), cisplatin (single dose, 7 mg/kg b.w., i.p. dissolved in 0.9% saline) and squalene (100 mg/kg squalene (dissolved in warm corn oil, daily orogastric gavage) cotreated (Cis/Squa). After 10 days, the mice were sacrificed under ketamine/xylazine anesthesia, blood samples were taken and the kidneys were dissected for histological and molecular analysis.

### 2.2. Oxidative stress analysis

Total antioxidant status (TAS) and total oxidant status (TOS) of blood were measured colorimetrically using RelAssay Diagnostics Kits (Gaziantep, Turkey) using an automated measurement method by an automated analyzer (Chromate Manager 4300, Palm City/USA). As described in a previous study (Kilic et al., 2015b), the measurement of TAS was performed by monitoring the rates of the Fenton reaction and it was expressed as in terms of mmol equiv/L Trolox (Rel Assay Diagnostics, Gaziantep, Turkey). Similarly, the measurement of TOS was performed by monitoring the intensity of the colored complex produced by the reaction of ferric ions with xylenol orange and it was expressed as in terms of micromolar hydrogen peroxide (H2O2) equivalents per liter (μmol H2O2 equiv/L). Using these TAS and TOS values, oxidative stress index (OSI) was calculated for each sample using the following formula (Shimeda et al., 2005): OSI = (TOS) / (TAS) ***×*** 1000) ***× ***100

### 2.3. Analyses of proinflammatory/antiinflammatory cytokines

Proinflammatory markers (interferon-gamma (IFNγ), Interleukin (IL)17 and tumor necrosis factor-alpha (TNF-α)) and antiinflammatory markers (IL6 and IL10) from the kidney tissue homogenates were measured using Magpix Luminex System (Austin, TX, USA). Briefly, samples were incubated with primary antibodies for each cytokine which were immobilized on magnetic beads overnight at 4 ***°***C. On the following day, the samples were incubated with streptavidin-phycoerythrein solution in dark at room temperature. They were then were measured luminometrically and the data were evaluated using XPONENT software. 

### 2.4. Western blot analysis

The total protein extraction was performed from homogenates of one of the kidney tissues of animals. Equal amounts of protein which were determined using Qubit Fluorometer 2.0 (Invitrogen, USA) were diluted in sample buffer, denatured, and loaded onto 4%-12% Bis-Tris polyacrylamide gels (Invitrogen). Proteins were transferred onto polyvinylidene fluoride (PVDF) membranes with iBlot® (Invitrogen) gel transfer system. Membranes were dried, incubated in blocking solution (5% milk powder in 0.1% Tween 20/0.1 M Tris-buffered saline (TBST)), and immersed with primary antibodies (anti-p-mTOR (mammalian target of rapamycin), anti-mTOR, anti-Akt (protein kinese B), anti-p-Akt, anti-GSK-3β (glycogen synthase kinase-3), anti-Nrf2) (Santa Cruz, CA, USA) in appropriate concentrations for incubation overnight at 4°C. On the following day, the secondary antibodies (horseradish peroxidase-linked goat antimouse IgG (immunoglobulin G), Abcam, Cambridge, UK) were used to incubate membrane for 1 h at room temperature. All blots were performed at least three times and they were developed by using Enhanced Chemiluminescence -Advanced Western Blotting Detection Kit (Amersham, UK). Proteins were visualized by Bio-Rad ChemiDoc XRS System (Bio-Rad Laboratories Inc., USA) and analyzed densitometrically using ImageJ software. The samples were normalized using a monoclonal mouse antibody against to β-actin (A5316; Sigma, USA). 

### 2.5. Histological evaluation

From the sacrificed animals, the other kidney tissues were taken and the entire tissue blocks were covered with cryo-embedding media to store the frozen tissue block at -80° C until ready for sectioning. The frozen tissue block was cut on a cryotome into 5-7 µm sections for histological examinations. The kidney sections were stained with periodic acid Schiff (PAS) using conventional methods for light microscopic examinations (Nikon Eclipse Ni research microscope). As described in one of our studies (Ayla et al., 2011), a semiquantitative score was developed to evaluate the degree of the damage by examining a minimum of 20 glomeruli (range 20 to 60) in each specimen. The scores obtained by the two investigators were averaged. Photographs of sections were taken at different magnifications in a Nikon Eclipse Ni research microscope, fitted with Nikon DS-Fi2 model digital camera and used Nikon Sight D5 V3 software. 

### 2.6. Data analysis and statistics

Statistical analysis was performed using SPSS 18.0 for Windows. Significance was evaluated by one-way ANOVA. The post hoc comparisons of simple effects were conducted using Fisher’s Least Significant Difference (LSD) test. The criterion of statistical significance was P ≤ 0.05. All values are given as mean ± S.D.

## 3. Results

### 3.1. Oxidative stress markers

**Figure 2:**
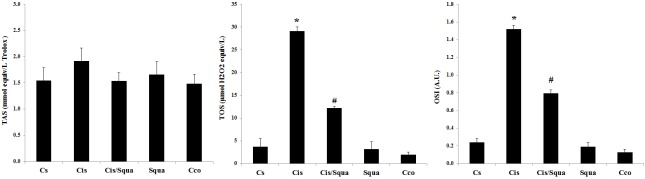
Levels of TAS, TOS, and OSI in Cs (control-received saline), Cis (cisplatin-treated), Cis/Squa (cisplatin and squalene cotreated), Squa (squalene-treated), and Cco (control receiving corn oil) groups in the blood serum. TOS and OSI levels decreased significantly in Cis/Squa group relative to the Cis group. (*) indicates the comparison with the Cs group, (#) indicates the comparison with the Cis group. The results are shown as mean ± standart error of mean (SEM). A.U., Arbitrary units.

To measure the antioxidative effect of squalene, we determined the TAS, TOS, and OSI levels for each group. Figure  2 shows that oxidative stress (both OSI and TOS levels) was found to be increased nearly 6-fold in the Cis group relative to the Cs group (P < 0.05), whereas OSI value decreased in the cisplatin and squalene co-treated (Cis/Squa) group (P < 0.05) (Figure  2). 

### 3.2. Proinflammatory and antiinflammatory markers

**Figure 3:**
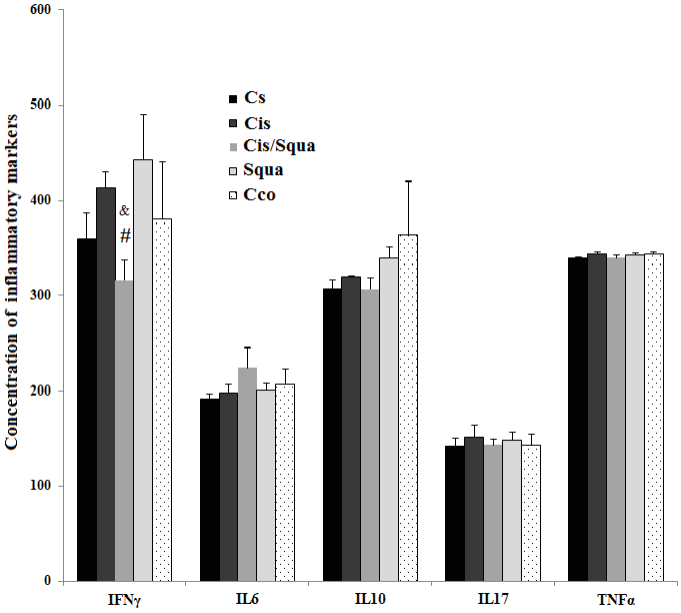
The concentrations of selected inflammatory markers (IFNγ, IL6, IL10, IL17, and TNF-α) in the kidney tissue homogenates which were measured using Magpix Luminex System in Cs (control-received saline), Cis (cisplatin-treated), Cis/Squa (cisplatin and squalene cotreated), Squa (Squalene-treated), and Cco (control receiving corn oil) groups. (#) indicates the comparison with the Cis (cisplatin-treated) group, (&) indicates the comparison with the Squa (squalene-treated) group. For statistical significance, P ≤ 0.05.

**Figure 4:**
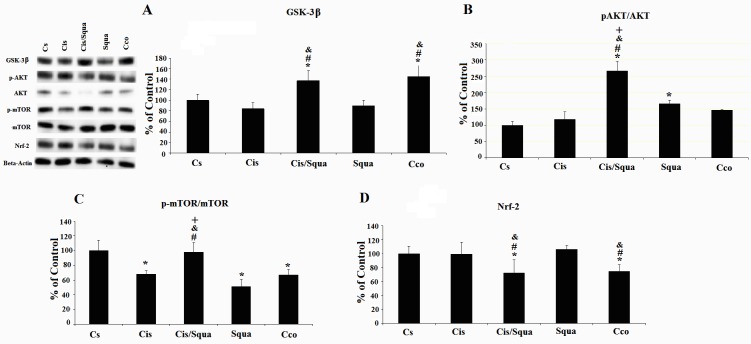
Western blot data of kidney tissue. (a) shows total GSK-3β, (b) p-Akt/total Akt, (c) p-mTOR/total mTOR, and (d) Nrf2. (*) indicates the comparison with the Cs (control-received saline) group, (#) indicates the comparison with the Cis (cisplatin-treated) group, (&) indicates the comparison with the Squa (squalene-treated) group, and (+) indicates the comparison with the Cco (control receiving corn oil) group, all data were normalized against β-actin. For statistical significance, P ≤ 0.05.

To investigate the effect of both cisplatin and squalene on the inflammation, we measured the concentration of proinflammatory and antiinflammatory markers in the kidney tissues (Figure  3). According to one-way ANOVA, there is a marginal difference between-groups in the level of IFN-γ (F(4,23) = 2.478, P = 0.079)(P = 0.038) and the squalene-treated group (P = 0.009) (Figure  3). However, in the other inflammatory markers, there was no treatment effect among the studied groups (IL-6, F(4,23) = 0.902, P = 0.482; IL-10, F(4,23) = 1.607, P = 0.214; IL-17, F(4,23) = 0.211, P = 0.929; and TNF-α, F(4,23) = 0.723, P = 0.587(Figure  4). The expression level of total GSK-3β was significantly increased in cisplatin and squalene cotreated mice and control mice receiving corn oil by gavage relative to the control mice receiving saline (37.32% and 45.50%, respectively; P < 0.05, Figure  4a). In addition, the expression level of total GSK-3β of cisplatin and squalene cotreated mice was significantly increased compared to that of the cisplatin-treated mice, by 62.75% (P < 0.05). Western blots of the ratio of phospho-Akt to total AKT were visualized in Figure  4b. According to the one-way ANOVA, there were significant treatment effects between groups (F(4,14) = 12.340, P = 0.001(Sgua group) (65.32%) or after cisplatin treatment (Cis/Squa group) (165.74%) increased the level of phospho-Akt compared to the control group (Cs grup) (P = 0.031 and P < 0.001, respectively). Meanwhile, the level of phospho-Akt to total Akt in cisplatin and squalene cotreated mice (Cis/Squa) was also higher than that of both in cisplatin treated mice (Cis) (148%) and control mice receiving corn oil (Cco) (120%) (P < 0.001) (Figure  4b). The levels of the ratio of phospho-mTOR to total mTOR proteins were also investigated (Figure  4c). According to the one-way ANOVA, there were also significant treatment effects between groups in the ratio of phospho-mTOR to the mTOR (F(4,14) = 14.684, P < 0.001(32.13%), squalene-treated mice (48.69%), and control mice receiving corn oil (33.01%) (P = 0.002, P < 0.001, and P = 0.002, respectively). The expression level of phospho-mTOR significantly increased in cisplatin and squalene co-treated mice compared to those of the cisplatin-treated mice, squalene-treated mice, and control mice receiving corn oil, by 30%-40% (P = 0.003, P < 0.001, and P = 0.003, respectively). When the expression level of Nrf2 was investigated by Western blotting, we observed a significant between-groups difference according to one-way ANOVA (F(4,14) = 4.152, P = 0.031(Cis/Squa) (27.2%) and mice receiving corn oil (Cco)(25.2%) relative to the control mice receiving saline (Cs) (P = 0.032 and P = 0.044, respectively) (Figure  4d). The very similar trend was observed when the levels of Nrf2 in cisplatin and squalene cotreated mice (Cis/Squa) and mice receiving corn oil (Cco) were compared with those of the cisplatin-treated mice (Cis) (P = 0.033 and P = 0.045, respectively) and squalene-treated mice (Squa) (P = 0.012 and P = 0.016, respectively).

### 3.4. Histopathological changes

**Figure 5:**
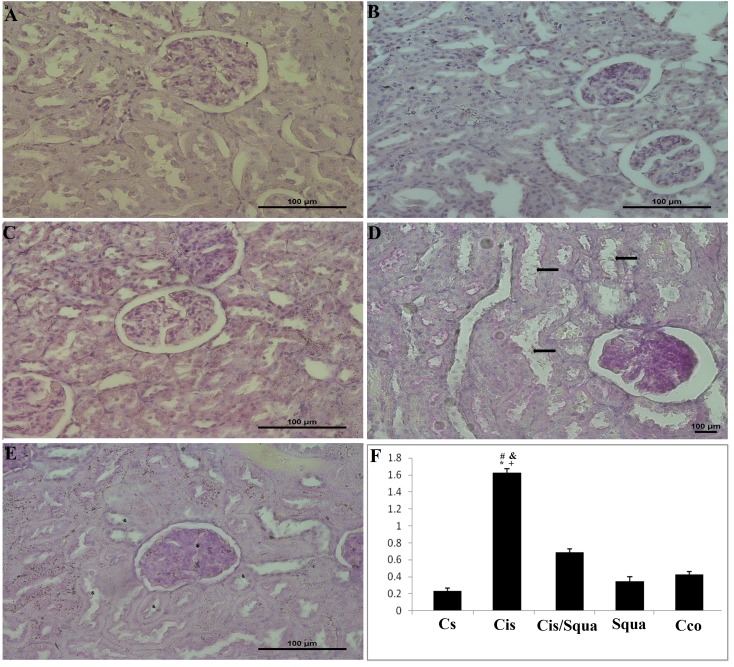
PAS staining was applied for identifying the effects of cisplatin exposure on the expansion of mezengial matrix scoring in kidney tissues of the rats. Matrix expansion (shown with *) and proximal and distal tubul damages (shown with à) were detectable in cisplatin groups (d). Treatment with squalene groups (e) resulted in almost normal tubules (shown with &) and matrix expansion (shown with *) less than cisplatin group. In control (Cs) group (a), corn oil (Cco) (b), and squalene group (c) histological structure were normal. Magnification, a–e: 40×. (f) Mesangial matrix expansion % tissue in the kidney glomeruli. (*) indicates the comparison with the Cs (control receiving saline) group, (#) indicates the comparison with the cisplatin group, (&) indicates the comparison with the squalene group, and (+) indicates the comparison with the Cco (control receiving corn oil) group, all data were normalized against β-actin. For statistical significance, P ≤ 0.05.

Histopathologically, there were no abnormal findings for the kidney of the control mice receiving saline (Cs), mice receiving corn oil (Cco), and squalene-treated mice (Squa) in the light microscopic examination (Figures 5a-5c). Degenerative changes were observed in the renal glomeruli and tubules of cisplatin-treated mice (Cis). Dilatation in the capillaries and urinary spaces were noted and the shape of the flat epithelial cells of the parietal layer of Bowman’s membrane was mostly cuboidal or round. In the proximal tubules, vacuolization and degeneration in the endothelial cell cytoplasm and loss in the microvillus structure were recorded (Figure  5d). Treatment with squalene ameliorated these changes as showing almost the same light microscopic appearance with controls in the tubules and glomeruli (Figure  5e). The graded histological changes (Mesangial matrix expansion) for each group are shown in Figure  5f. According to one-way ANOVA, there was a significant difference in the histopathological damage score (H) score among groups (F(4,30) = 165,4, P ≤ 0.001(P ≤ 0.001). However, squalene administration decreased this H score significantly in the cisplatin and squalene cotreated mice compared to cisplatin-treated mice (P ≤ 0.001). 

## 4. Discussion

Cisplatin is a well-known anticancer drug and it is especially effective in certain types of cancers, for example bladder, ovarian, and testicular carcinomas (Dasari and Tchounwou, 2014). The mechanism of action of cisplatin is very much similar to those of the other anticancer agents; it directly interferes with the DNA replication machinery of the cell by making crosslinks. Through these crosslinks, cisplatin inhibits the cell to enter into mitotic cycle, thereby cell proliferation is halted, and the apoptotic program is activated. Although cisplatin’s therapeutic effect significantly increases at high doses, its clinical use is limited due to serious side effects such as nephrotoxicity because kidneys are one of the main sites where toxins are concentrated in body due to high blood flow. Nephrotoxic effect of cisplatin can reduce glomerular filtration rate and cause serum electrolyte disturbances and permanent kidney failure. Its toxic effects depend largely on oxidative stress induction, and the prophylactic use of antioxidants could sometimes help to decrease toxicity. Therefore, our study indicated that the nephrotoxic effects of cisplatin could be attenuated by the use squalene with its antioxidant and prosurvival effects.First, our results clearly indicated that cisplatin administration boosted the oxidative stress and application of squalene reduced this oxidative stress. This is consistent with the literature of cisplatin toxicity (Verma et al., 2016) and the OSI data would provide a support for the use of squalene as an antioxidant in chemotherapeutic applications of cisplatin. Previously, it was suggested that squalene exerts its anticarcinogenic activity by acting as an antioxidant to enhance cellular antioxidant status (Hashim et al., 2005; Smith, 2010). In addition, squalene inhibits oxidative stress by reducing levels of reactive oxygen species to protect against oxidative DNA damage in human breast cancer (Warleta et al., 2010). Although cisplatin exerted a definite oxidative stress on the kidneys in our experimental model, renal tissue did not exhibit any statistically significant change in the inflammatory markers except IFN-γ. Based on these findings, it could be considered that the inflammation-related toxic effect of cisplatin on kidneys occurs through IFN-γ, as previously observed in the study by Kimura et al. 2012. The decrease in the IFN-γ level by the administration of squalene after cisplatin treatment showed that squalene may take its antioxidative or antiinflammatory effect through inhibiting the IFN-γ expression in the kidney cells. Therefore, these results clearly showed that cisplatin treatment greatly induced the levels of OSI and IFN-γ in our experimental animals and squalene administration ameliorated the oxidative stress and inflammation.It is well known that Nrf2 is a transcription factor, and its expression is induced when cellular damage occurs via oxidative stress, to regulate the adaptive response to oxidants and electrophiles (Ma, 2013). When a chemopreventive agent is given to the organism, pharmacological boosting of the Nrf2 activity occurs to protect the organism from oxidative damage that may occur due to toxic metabolites or diseases such as cancer, mitochondrial damage, or ethanol-induced lesions (Talalay et al., 2003; Dong et al., 2008; He and Ma, 2012). However, in our experimental model, Nrf2 expression significantly decreased in cisplatin and squalene cotreated mice relative to both control (Cs) and cisplatin-treated mice. In a previous study, investigators also found that dietary squalene supplementation did not change the expression of Nrf2 protein in DSS-induced acute colitis (Sánchez-Fidalgo et al., 2015). Using the Western blot, we also investigated the activation of AKT to observe the effect of squalene administration on cell survival after cisplatin treatment (Fu et al., 2016). In our study, a significant increase in the phosphorylated AKT was observed in the squalene treatment suggesting the cell survival enhancing effect of squalene. This effect was more efficient where there was a damaging factor such as cisplatin treatment, in our case. In addition, the detrimental effect of cisplatin was especially observed in the phosphorylation of mTOR protein. Cisplatin induced inactivation of mTOR also observed in the previous studies (Liu et al., 2018). It is generally known that phosphorylated (activated) mTOR in the complex of mTORC2 phosphorylates and activates AKT. Similarly, the administration of squalene ameliorates the effect of cisplatin on the phosphorylation of mTOR in the kidney cells suggesting the action mechanism of squalene through AKT/mTOR signaling pathway. Interestingly, it is noted that squalene at very high doses (1%) exerts the chemopreventive effect due to the HMG-CoA reductase inhibition (Rao et al., 1998). As it was known, HMG-CoA reductase inhibition attenuates AKT/mTOR signaling and induces autophagy. In a previous in vitro study, it was observed that using statins which are inhibitors of HMG-CoA reductase impairs the phosphorylation of AKT by activating mTOR and inhibiting forkhead box O (FoxO) transcription factors (Bonifacio et al., 2015). In addition, inhibition of HMG-CoA reductase inhibits lipid-anchoring of the small G protein Rac1 to perturb mTOR signaling which has been identified as a key modulator of autophagy for more than a decade (Wei et al., 2013; Paquette et al., 2018). However, the dosage of squalene that we used increased the phosphorylation of AKT and mTOR to behave as a prosurvival molecule. On the other hand, a significant increase in total GSK-3β was observed in cisplatin and squalene cotreated mice. The data about GSK-3β may be interpreted with the AKT data, because it is known that activation of AKT by phosphorylation, which is one of the main prosurvival proteins in the cell, inhibits GSK-3β phosphorylation and stimulation suggesting an increase in the total GSK-3β expression without affecting the expression of phosphorylated GSK-3β (Hermida et al., 2017). These findings indicate that inactive GSK-3β could not stimulate apoptosis in kidneys; therefore, squalene can promote the survival of renal cells which may protect the cells from cisplatin’s toxic effects. In addition, the same effect was observed in the corn oil group suggesting that the increase in the expression of GSK-3β in the cisplatin and squalene cotreated mice was independent from the effects of squalene per se.The molecular and cellular mechanisms of cisplatin-induced renal cell death have been studied over the past 10 years. Among these mechanisms, inflammation occurring due to injury of renal epithelial cells had gained importance because of its capacity to amplify kidney injury and dysfunction in vivo**(Arany and Safirstein, 2003; Sastry and Kellie, 2005). A recent report indicated that cisplatin therapy leads to the dilatation, vacuolization, and loss of tubular epithelial cells; and glomerular degeneration and edema in the kidney tissues, as seen in our study (Eren et al., 2018). The previous findings and our results also confirmed that cisplatin produces high H score in the kidney (Ozkok and Edelstein, 2014; Eren et al., 2018; Soyman et al., 2018;). In the literature, these histopathological findings point out the nephrotoxicity (Yao et al., 2007). In the current study, squalene ameliorated nephrotoxicity occurring due to cisplatin toxicity in mouse kidney, as evidenced by lower H score. Treatment with squalene resulted in almost normal tubules and glomeruli in the light microscopic examination. The cause of nephrotoxicity is thought that cisplatin accumulates in the proximal tubular inner medulla and outer cortices of kidney after quick filtration through the glomerular basal membrane and it results in tubular cell death (Kulmann et al., 1997). The underlying mechanisms of tubular cell death due to cisplatin treatment includes oxidative stress, DNA adducts, inflammation, mitochondrial dysfunction, apoptosis, and direct cytotoxicity (Yao et al., 2007). However, several studies showed the protective effects of antioxidative molecules against cisplatin-induced damages (Eren et al., 2018; Haghi-Aminjan et al., 2018; Soyman et al., 2018). Therefore, we may suggest that squalene treatment ameliorated the cisplatin-induced histopathological damages in the kidney tissue by regulating the balance of the redox system due to its antioxidative effect (Ondrejková et al., 2017). In conclusion, our study results are the first to indicate that squalene offers a protective effect against cisplatin-induced nephrotoxicity in mice, by attenuating the oxidative stress. Squalene activates the AKT/mTOR signaling pathway and reduces histopathological alterations in cisplatin-induced kidney damage. Therefore, squalene administration may be a promising approach in protecting kidney tissue from the detrimental effects of cisplatin due to its antioxidative effect.

## Contribution of authors

The first two authors had equal contribution. Ü.K. and R.K. were involved in planning and supervised the work. A.S., M.O., Y.Y., and H.B. M.Y.G. performed the measurements. A.S., M.O., B.E., K.S., and Ü.K. processed the experimental data, performed the analysis, and designed the figures. S.A. performed and analyzed the histological data. U.K. and B.E. drafted and finalized the manuscript. All the authors discussed the results and commented on the manuscript.

## References

[ref1] (2003). Cisplatin nephrotoxicity. Seminars in Nephrology.

[ref2] (2011). Doxorubicin induced nephrotoxicity: protective effect of nicotinamide. International Journal of Cell Biology.

[ref3] (2015). The AKT/mTOR signaling pathway plays a key role in statin-induced myotoxicity. Biochimica et Biophysica Acta.

[ref4] (2008). Squalene selectively protects mouse bone marrow progenitors against cisplatin and carboplatin-induced cytotoxicity in vivo without protecting tumor growth. Neoplasia.

[ref5] (2014). Cisplatin in cancer therapy: molecular mechanisms of action. European Journal of Pharmacology.

[ref6] (2008). Nrf2-mediated transcriptional induction of antioxidant response in mouse embryos exposed to ethanol in vivo: implications for the prevention of fetal alcohol spectrum disorders. Antioxidants and Redox Signaling.

[ref7] (2018). Whortleberry protects kidney against the cisplatin-induced nephrotoxicity: an experimental study. Renal Failure.

[ref8] (2017). Enhanced protection of C57 BL/6 vs Balb/c mice to melanoma liver metastasis is mediated by NK cells. Oncoimmunology.

[ref9] (2016). Shikonin inhibits inflammation and chondrocyte apoptosis by regulation of the PI3K/Akt signaling pathway in a rat model of osteoarthritis. Experimental and Therapeutic Medicine.

[ref10] (2018). The protective role of melatonin in chemotherapy-induced nephrotoxicity: a review of non-clinical studies. Expert Opinion on Drug Metabolism and Toxicology.

[ref11] (2005). Components of olive oil and chemoprevention of colorectal cancer. Nutrition Reviews.

[ref12] (2017). GSK3 and its interactions with the PI3K/AKT/mTOR signalling network. Advances in Biological Regulation.

[ref13] (2012). Disruption of Nrf2 synergizes with high glucose to cause heightened myocardial oxidative stress and severe cardiomyopathy in diabetic mice. Journal of Diabetes and Metabolism 2012 (suppl 7).

[ref14] (2003). Platinum-based anticancer agents: innovative design strategies and biological perspectives. Medicinal Research Reviews.

[ref15] (2013). Melatonin suppresses cisplatin-induced nephrotoxicity via activation of Nrf-2/HO-1 pathway. Nutrition and Metabolism (Lond) 10 (1): 7.

[ref16] (2015a). Enhancement of cisplatin sensitivity in human cervical cancer: epigallocatechin-3-gallate. Frontiers in Nutrition.

[ref17] (2015b). A remarkable age-related increase in SIRT1 protein expression against oxidative stress in elderly: SIRT1 gene variants and longevity in human. PLoS One.

[ref18] (2012). Interferon-γ is protective in cisplatin-induced renal injury by enhancing autophagic flux. Kidney International.

[ref19] (1997). Insights into potential cellular mechanisms of cisplatin nephrotoxicity and their clinical application. Nephrology Dialysis Transplantation.

[ref20] (2017). The efficacy and safety of gemcitabine, cisplatin, prednisone, thalidomide versus CHOP in patients with newly diagnosed peripheral T-cell lymphoma with analysis of biomarkers. British Journal of Haematology.

[ref21] (1976). Measurement of squalene in human tissues and plasma: validation and application. Journal of Lipid Research.

[ref22] (2018). Histone deacetylase inhibitors protect against cisplatin-induced acute kidney injury by activating autophagy in proximal tubular cells. Cell Death and Disease.

[ref23] (2013). Role of Nrf2 in oxidative stress and toxicity. Annual Review of Pharmacology and Toxicology.

[ref24] (2017). Cisplatin-Induced Nephrotoxicity; Protective Supplements and Gender Differences. Asian Pacific Journal of Cancer Prevention.

[ref25] (1997). Squalene, olive oil, and cancer risk: a review and hypothesis. Cancer Epidemiology Biomarkers and Prevention.

[ref26] (1999). Squalene, olive oil, and cancer risk. Review and hypothesis. Annals of the New York Academy of Sciences.

[ref27] (2017). Antioxidative protection of squalene adjuvant and rabies vaccine with adjuvant. Biological and Pharmaceutical Bulletin.

[ref28] (2014). Pathophysiology of cisplatin-induced acute kidney injury. Biomed Research International.

[ref29] (2018). mTOR pathways in cancer and autophagy. Cancers.

[ref30] (2013). Evidence against protective role of sex hormone estrogen in Cisplatin-induced nephrotoxicity in ovarectomized rat model. Toxicology International.

[ref31] (1998). Chemopreventive effect of squalene on colon cancer. Carcinogenesis.

[ref32] (2015). Dietary squalene supplementation improves DSS-induced acute colitis by downregulating p38 MAPK and NFkB signaling pathways. Molecular Nutrition and Food Research.

[ref33] (2005). Severe neurotoxicity, ototoxicity and nephrotoxicity following high-dose cisplatin and amifostine. Pediatric Hematology and Oncology.

[ref34] (2006a). Effect of squalene on cyclophosphamide-induced toxicity. Clinica Chimica Acta.

[ref35] (2006b). Attenuation of cyclophosphamide induced toxicity by squalene in experimental rats. Chemico-Biological Interactions.

[ref36] (2005). Protective effects of capsaicin against cisplatin-induced nephrotoxicity in rats. Biological and Pharmaceutical Bulletin.

[ref37] (1997). Ebselen as a glutathione peroxidase mimic and as a scavenger of peroxynitrite. Advances in Pharmacology.

[ref38] (2000). Squalene: potential chemopreventive agent. Expert Opinion on Investigational Drugs.

[ref39] (2008). Anticarcinogenic compounds of olive oil and related biomarkers. European Journal of Nutrition.

[ref40] (2018). Can ebselen prevent cisplatin-induced ovarian damage?. Archieves of Gynecology and Obstetrics.

[ref41] (2003). Importance of phase 2 gene regulation in protection against electrophile and reactive oxygen toxicity and carcinogenesis. Advances in Enzyme Regulation.

[ref42] (2008). Supercritical carbon dioxide fractionation of nonesterified alkoxyglycerols obtained from shark liver oil. Journal of Agricultural and Food Chemistry.

[ref43] (2016). Total antioxidant and oxidant status of plasma and renal tissue of cisplatin-induced nephrotoxic rats: protection by floral extracts of Calendula officinalis Linn. Renal Failure.

[ref44] (2005). Cellular processing of platinum anticancer drugs. Nature Reviews Drug Discovery.

[ref45] (2010). Squalene protects against oxidative DNA damage in MCF10A human mammary epithelial cells but not in MCF7 and MDAMB- 231 human breast cancer cells. Food and Chemical Toxicology.

[ref46] (2013). Enhancement of autophagy by simvastatin through inhibition of Rac1-mTOR signaling pathway in coronary arterial myocytes. Cell Physiology and Biochemistry.

[ref47] (2007). Cisplatin nephrotoxicity: a review. The American Journal of The Medical Sciences.

